# Radiographic Characteristics of the Femoral Nutrient Artery Canals in Total Hip Arthroplasty using Cementless Femoral Stem

**DOI:** 10.5704/MOJ.2303.015

**Published:** 2023-03

**Authors:** YH Roh, SJ Yoo, TH Choi, KW Nam

**Affiliations:** 1 Department of Orthopaedic Surgery, Jeju National University Hospital, Jeju City, South Korea; 2 Department of Orthopaedic Surgery, Uijeongbu Eulji Medical Center, Uijeongbu, South Korea

**Keywords:** hip arthroplasty, nutrient artery canal, periprosthetic femoral fracture, cementless stem, radiographic study

## Abstract

**Introduction:**

Accurate diagnosis of undisplaced periprosthetic femoral fracture (PFF) after hip arthroplasty is crucial, as overlooked PFF may affect its treatment and prognosis. The undisplaced PFF is often difficult to distinguish from radiolucent lines of nutrient artery canal (NAC) of the femur present on post-operative radiographs. We aimed to identify the radiographic features of NAC to distinguish them from PFFs.

**Materials and methods:**

In this retrospective radiological study, a total of 242 cases in 215 patients with hip arthroplasty were analysed using pre-operative and post-operative anteroposterior (AP) and translateral (TL) radiographs. Interobserver agreement of the measurements was assessed by two independent experienced orthopaedic surgeons. The kappa value ranged from 0.83 to 0.87, indicating strong agreement according to the Landis and Koch criteria.

**Results:**

The NACs were found pre-operatively in 94 (39.8%) cases on AP views and in 122 cases (50.4%) on TL views. The radiolucent lines were observed post-operatively in 42 (17.4%) on AP views and 122 (50.4%) on the TL views. three cases (1.2%) had a fracture around the stem that were detected on radiographs. One case with PFF presented simultaneously with NAC on the immediate post-operative radiographs. All patients were treated by conservative measures, and the radiolucent lines did not appear on follow-up radiographs.

**Conclusion:**

It is not easy to differentiate undisplaced PFFs that can occur after hip arthroplasty operation from NACs. However, accurate diagnosis is possible through careful observation and comparison of pre-operative and post-operative radiologic images.

## Introduction

In cementless total hip arthroplasty (THA), it is important to have a rigid fixation at the interface between the bone of the femur and the stem. However, when the stem is forcefully inserted to obtain rigid fixation, fracture on the femur may occur around the stem. Moreover, surgery using minimally invasive technique, anatomical mismatch between femur and stem, severe osteoporosis and revision arthroplasty increase the risks of intra-operative periprosthetic femoral fracture (PFF)^1.^ Although PFF occurring at the proximal metaphysis is easily detected intra-operatively, undisplaced diaphyseal PFF caused by impact of the stem tip to the femur shaft is challenging to diagnose and incidentally detected on post-operative plain radiographs. The intra-operative PFF in the primary cementless THA has been reported to be at 1% to 5.4%, and its incidence even reaches 10.9% to 19% in revisional arthroplasty^2^.

Undisplaced PFFs are not prominent on post-operative plain radiographs, and the diagnosis is often delayed due to nutrient artery canal (NAC). Conversely, NACs may be misdiagnosed as fracture, leading to unnecessary treatment. Therefore, we aimed not only to evaluate radiological analysis of the NAC but also to identify differential radiological features associated with undisplaced PFF in patients who underwent total hip joint replacements.

## Materials and Methods

We reviewed 312 cases in 272 patients with cementless THA or hip hemiarthroplasty from March 2012 to April 2017. We included only cementless femoral stems which are long enough to reach the linea aspera, known to be the entry point of the nutrient artery. The selected cementless stems were Versys [Zimmer®, Warsaw, Indiana, USA] in 112 cases and Summit [DePuy®, Raynham, Massachusetts, USA] in 200 cases, stem lengths of which were 110~155mm and 125~170mm, respectively. Four cases with pathological fractures, 16 cases with femur deformations, 40 cases of revisions and 10 cases with definite fractures detected intra-operatively were excluded. Therefore, 242 cases in 215 patients were enrolled in this study. The Jeju National University Hospital institutional review board approved.

**Fig. 1:**
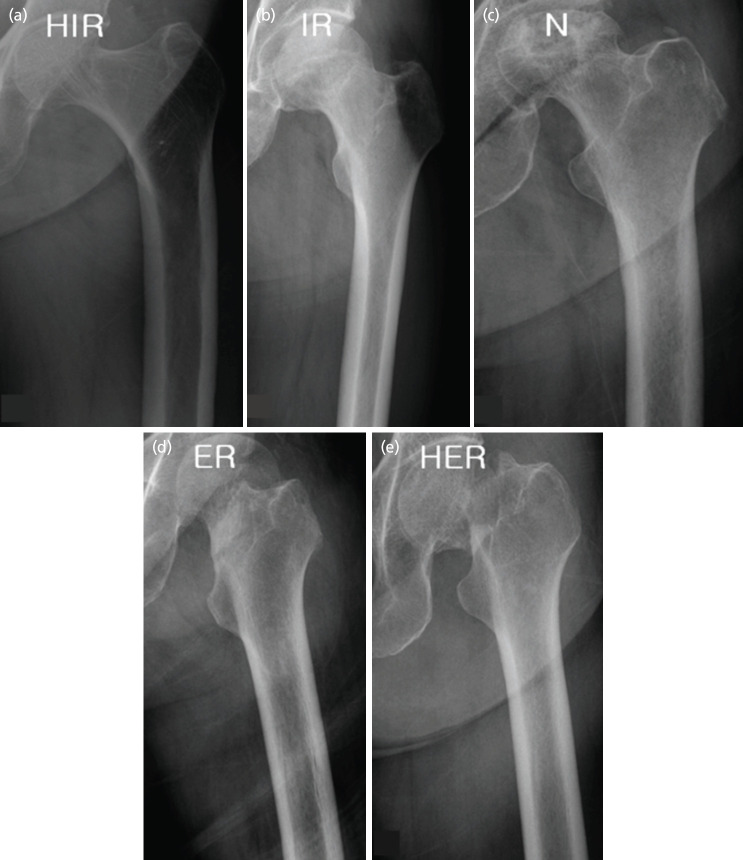
Classification by rotation of the femur. (a) Hyper internal rotation (HIR) group. (b) Internal rotation (IR) group. (c) Neutral (N) group. (d) External rotation (ER) group. (e) Hyper external rotation (HER) group.

Pre-operative and immediate post-operative anteroposterior (AP) and translateral (TL) views were taken for analysis. Pre-operative plain radiographs were examined for the presence of NAC. When the canals were visible, they were classified into five groups according to degrees of rotation of the femur, as described in Zhang’s study of degree of rotation based on ratios of height and width of lesser trochanter^3^. Internal rotation exceeding 15° was classified as hyper internal rotation (HIR) group; between 5° to 15° of internal rotation as internal rotation (IR) group; internal and external rotation of 5° or less as neutral (N) group; between 5° to 15° of external rotation as external rotation (ER) group; and external rotation exceeding 15° as hyper external rotation (HER) group (Fig. 1).

**Fig. 2:**
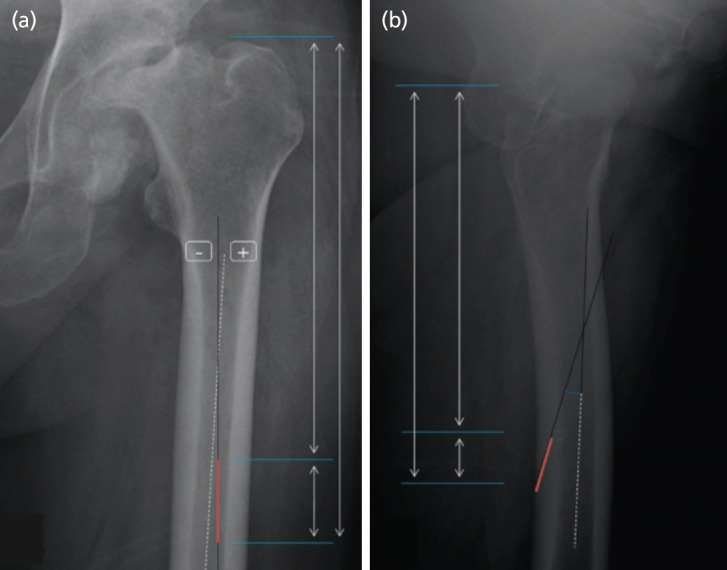
Radiographic measurement of the nutrient artery canal. (a) Anteroposterior view. (Bold red line: nutrient artery canal, Dotted white line: anatomical axis of femur, +: positive value of angle, -: negative value of angle, Inner superior white arrow line: distance from the tip of the greater trochanter to the proximal end of nutrient artery canal, Inner inferior white arrow line: length of nutrient artery canal, Outer white arrow line: distance from the tip of the greater trochanter to the distal end of nutrient artery canal). (b) Translateral view. (Bold red line: nutrient artery canal, Dotted white line: central line of femur. The rest indicate the same things of anteroposterior view).

Distance from the tip of the greater trochanter to the proximal and distal ends of the canals were measured in AP views to evaluate the position and length of the canals^4^. The angle of the NACs was measured in respect to the anatomical axis of femur. A positive value was given if the proximal end was directed to the lateral side, and a negative value was given if the proximal end was intended for the medial side (Fig. 2a). In TL views, the position and length of the canals were evaluated in the identical manners as in AP^4^. The angle between the femoral shaft and the NACs were evaluated by measuring the angle of the canals in respect to a central line drawn from two central points of the femoral shaft at proximal 20mm to distal 20mm of the canal (Fig. 2b).

**Fig. 3:**
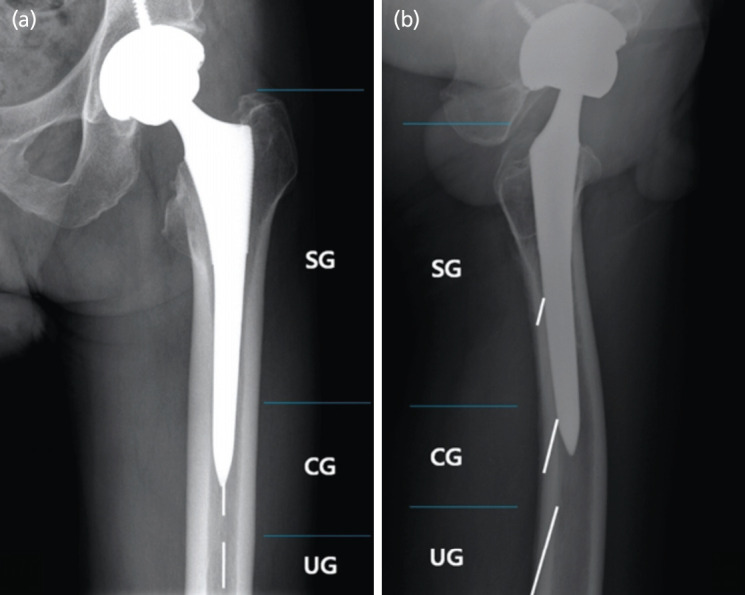
Classification by location of the radiolucent line, visible on post-operative radiographs. (a) Anteroposterior view. (Radiolucent line was above the stem tip: Suspected group (SG), Stem tip was in between both ends of the radiolucent line: Connected group (CG), The radiolucent line was located below the stem tip: Unconnected group (UG)). (b) Translateral view. (Using the same measurement methods of anteroposterior view).

Post-operative plain radiographs were classified according to the location of the radiolucent lines. In both AP views and TL views, they were categorised into three groups; radiolucent line above the stem tip as suspected group (SG), the stem tip in between both ends of the line as connected group (CG) and the line located below the stem tip as unconnected group (UG) (Fig. 3).

The plain radiographic equipment used in this study was a fluorospot compact FD [Siemens®, Munich, Bayern, Germany]. Hip anteroposterior and translateral views were taken by an experienced radiographer, adhering to the established modality. The peak kilovoltage and milliampere seconds, which are parameters affecting the plain radiograph penetration, averaged about 75-80 and 5-9, respectively. The captured images are transmitted to the picture archiving and communication system [PACS; INFINITT, Infinitt Healthcare®, Seoul, Korea], and detailed analysis is possible because the window width and window level can be adjusted. Interobserver agreement of the measurements was assessed by two independent experienced orthopaedic surgeons. The kappa value ranged from 0.83 to 0.87, indicating strong agreement according to the Landis and Koch criteria.

## Results

Out of the 242 cases, 97 cases were males, and 145 cases were females. The average age of patients was 68.2 years old (27-95 years old). Cause for the surgery include femur neck fracture (121 cases; 50.0%), osteonecrosis of femoral head (84 cases; 34.7%), osteoarthritis (31 cases; 12.8%), intertrochanteric fracture (3 cases; 1.2%), and non-union (3 cases; 1.2%).

On the pre-operative AP views, NACs were observed in 94 (38.8%) of 242 cases, and six of them (2.5%) were observed with two canals. On TL views, the canals were observed in 122 cases (50.4%), and 14 of them (5.8%) had two canals. In 63 cases (26.0%), NACs were observed in both AP and TL views. In 153 cases (63.2%), the canals were visible in either view (Table I).

**Table I:** Number of cases with visible nutrient artery canal on pre-operative plain radiographs.

	AP view			TL view	
	94 / 242 (38.8%)			122/ 242 (50.4%)	
1 NAC	2 NACs	Total	1 NAC	2 NACs	Total
88 (93.6%)	6 (6.4%)	94	108 (88.5%)	14 (11.5%)	122
**Only AP**		**Both AP and TL**		**Only TL**	
31 (12.8%)		63 (26.0%)		59 (24.4%)	

Abbreviations – AP: anteroposterior, TL: translateral, NAC: nutrient artery canal

On AP views, HIR, IR, N, ER and HER group were detected with visible NACs in 13 cases (13.8%), 12 cases (12.8%), 10 cases (10.6%), 23 cases (24.5%), and 36 cases (38.3%), respectively. On this view, average distance from the tip of the greater trochanter to the proximal end of the canal was 161 ± 36mm and to the distal end was 187 ± 42mm. The length of the canal averaged in 27 ± 11mm. The average angle between femur anatomical axis and the NACs was measured 1.4° ± 5.9°.

On TL views, the average distance from the tip of the greater trochanter to the proximal end of the canal was 156 ± 32mm, to the distal was 194 ± 42mm. The average length of the canal was measured 41 ± 24mm. The direction of the NAC was all directed towards the proximal through the posterior cortex. The average angle between femur central line and the NACs was measured 14.0° ± 7.5°.

**Fig. 4:**
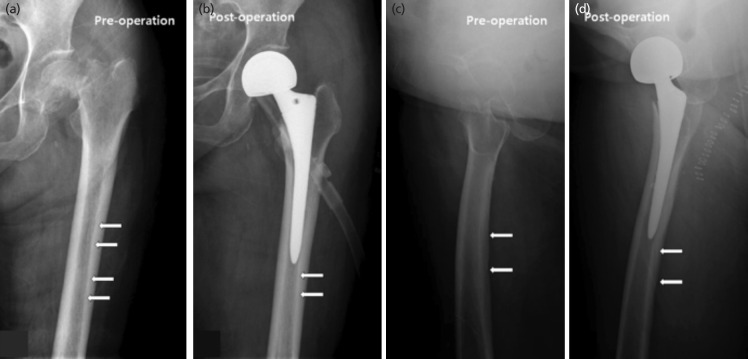
Radiolucent line at the same position as on pre-operative and post-operative radiographs. (a) Nutrient artery canal (arrow) on pre-operative anteroposterior view. (b) Radiolucent line (arrow) on post-operative anteroposterior view. (c) Nutrient artery canal (arrow) on pre-operative translateral view. (d) Radiolucent line (arrow) on post-operative translateral view.

In 42 (17.4%) of 242 case, radiolucent lines were observed on post-operative AP views. One of which (2.4%) was suspected group, 20 (47.6%) were connected group and 21 (50.0%) were unconnected group. In 28 (66.7%) of the 42 cases, we observed NAC at the same position as on pre-operative AP view (Table II) (Fig. 4a, b).

**Table II:** Number of cases with visible nutrient artery canals on post-operative anteroposterior view.

		AP view		
		42 / 242 cases (17.4%)		
SG	CG		UG	Total
1(2.4%)	20 (47.6%)		21 (50.0%)	42
		Visible NAC on pre-operative AP view		
		28 / 42 (66.7%)		

Abbreviations – AP: anteroposterior, SG: suspected group, CG: connected group, UG: unconnected group, NAC: nutrient artery canal

On the TL views, total of 133 radiolucent lines were observed in 122 (50.4%) of 242 cases. A total of 30 radiolucent lines (22.5%) were suspected group, 44 (33.1%) lines were connected group, and 59 (44.4%) lines were unconnected group. Eleven cases were seen with two radiolucent lines. In 95 (77.9%) of the 122 cases, there were NACs at the same position as on pre-operative radiograph (Table III) (Fig. 4c, d).

**Table III:** Number of cases with visible nutrient artery canals on post-operative translateral view.

		TL view		
		122 / 242 cases (50.4%) and 133 radiolucent lines		
SG	CG		UG	Total
30 (22.5%)	44 (33.1%)		59 (44.4%)	133
		Visible NAC on pre-operative TL view 95 / 122 (77.9%)		

Abbreviations – TL: translateral, SG: suspected group, CG: connected group, UG: unconnected group, NAC: nutrient artery canal

## Discussion

Fractures around the stem have been classified by several researchers based on the location and shape of the fracture. Vancouver classification is widely accepted because it includes locations of fracture, stem stability, and extent of bone loss in addition to suggested treatment guidelines^5-7^. Since nutrient artery foramen is mostly present in the middle 1/3 of femur diaphysis^8,9^, Vancouver type B1, B2 are the undisplaced intra-operative PFF that require differentiation from the NACs. Undisplaced PFFs occurring intra-operatively are often difficult to be identified not only during operation but also on post-operative plain radiographs. Schwartz and Engh reported 3% occurrence rate of the intra-operative PFF during cementless THA, and the half of them were not detected during operation^10^.

Nutrient artery originates from the second branch of the three perforating arteries of the deep femoral artery, and it enters through the linea aspera into the medullary canal^11,12^. Many studies have reported the presence of one or more nutrient artery foramens^13-15^. In a cadaveric study, Yamamoto *et al* found one nutrient artery in 55% and two nutrient arteries in 45% of the femur^12^. Imre *et al* reported a study using multi-detector computed tomography (CT) showing existence of at least one NAC and an average of two NACs^9^. Another anatomical study by Farouk *et al* described an existence of the nutrient artery foramen at an average of 161 ± 27mm below the tip of the greater trochanter^16^. Schiessel’s radiographic study measured an average distance of 170 ± 25mm from the greater trochanter tip to the proximal end of the NAC on AP views and 167 ± 27mm on TL views^4^. In our study, average distance from greater trochanter tip to the proximal end of the NAC, measuring 161 ± 36mm on AP views and 156 ± 32mm on TL views, showed no difference from previous studies.

In addition, it is observed in AP view that the NACs start from the posterior cortex and drive upward almost parallel to the femoral anatomical axis at an angle of 1.4° ± 5.9°, but in TL view it is observed to drive upward anterior at an angle of 14.0° ± 7.5° with the central line. Imre *et al* described 95% NACs traveling forward to upward direction, as seen in the current study^9^. It is more meaningful to evaluate the distance of the proximal end rather than the distal end of the NACs because intra-operative PFF is caused mainly by excessive hoop stress of endosteal cortex during rasp or implant insertion^5-7^.

**Fig. 5:**
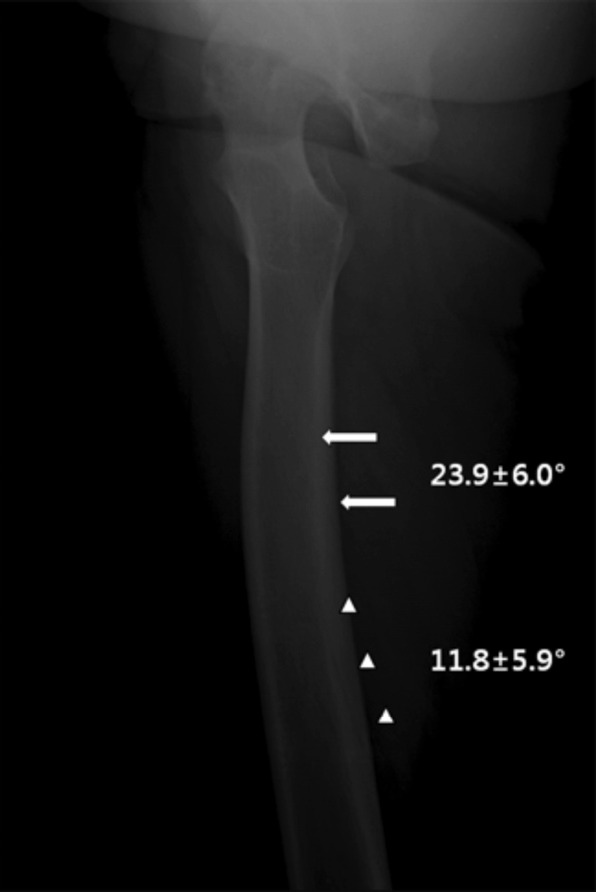
Two Nutrient artery canals are visible in the translateral view. Difference in nutrient artery canal slope angle. Proximal nutrient artery canal (arrow), distal nutrient artery canal (arrowhead).

In this study, the most frequently observed NACs in pre-operative AP view was the HER group, in which femur was over 15° externally rotated. However, it is critical not to confuse with that most NACs are observed with externally rotation because femur tends to be placed in the externally rotated position in order to lower the pressure of the hip joint in patients requiring total hip replacement. According to several previous studies, in some cases, the nutrient artery foramen is located on either medial or lateral side, not in between the medial and lateral lips of linea aspera^8,9^. Consequently, depending on the degree of rotation of the femur, NAC may not be observed in the pre-operative AP view. In the Schiessel’s radiological study, NACs were observed in AP views 15.5% and TL views 17.9%4. In our study, 38.8% were observed in AP view and 50.4% in TL view. This difference in NAC detection rate is considered to be due to the development of radiography equipment compared to the past. More than single NACs were observed in six cases (6.4%) on AP views and 14 cases (11.5%) on TL views. Interestingly in the TL views, the more proximal canals formed a gentle slope at an average of 23.9° ± 6.0°, the more distal canals averaged at a steeper angle of 11.8° ± 5.9°. This suggests that each nutrient artery may have originated from different nutrient artery foramens (Fig. 5).

There are cases where NAC was detected in the radiograph before surgery but not after surgery. When the NAC detected in the AP view before surgery was located relatively proximal, after surgery, it was hidden by the femoral stem in some cases. In particular, when there is no gap between the medulla and cortex due to tight insertion of the femur stem, or when the medullary space is narrow, such as a Dorr type A femur, it was not detected on post-operative AP view. In the TL view, when there is NAC in the cortex area on the pre-operative radiograph, NAC was observed in most cases in the post-operative radiograph. But NAC in the medullary portion was not observed well on the post-operative radiograph in some cases. In particular, it was difficult to observe on the radiograph after surgery in patients with a narrow AP diameter of the femur. NAC, which was not detected on the pre-operative radiograph, was observed in a total of 27 cases on the post-operative radiograph. In all cases, the femur was excessively rotated on the pre-operative radiograph due to severe necrosis of the femoral head, arthritis, or displacement of the fracture fragment.

In post-operative radiographs, those requiring watchful attention are suspected group and connected group, where radiolucent lines are connected to or are on the proximal side of stem tip. Yoon *et al* reported that Vancouver type B was 65% of the total PFF incidence, and in particular, Vancouver type B1 and B2 occurred frequently during operation and immediately after surgery^17^. In our study, suspected group and connected group are relatively common in AP views, with one (2.4%) and 20 (47.6%), respectively. In the TL views, suspected group 30 (22.5%) and connected group 44 (33.1%) were observed. The radiolucent lines belonging to the connected group should not be overlooked as NACs because it is observed around the stem and must be differentiated from PFFs. Post-operative AP views have a high rate of radiolucent lines being masked by stem. The longer and bigger the stem is, the more likely it is to cause challenges in differentiation^4^. Therefore, because the AP view alone is insufficient, the TL view should be supplemented, and, if necessary, additional radiographic evaluation of rotated femur may be helpful.

**Fig. 6:**
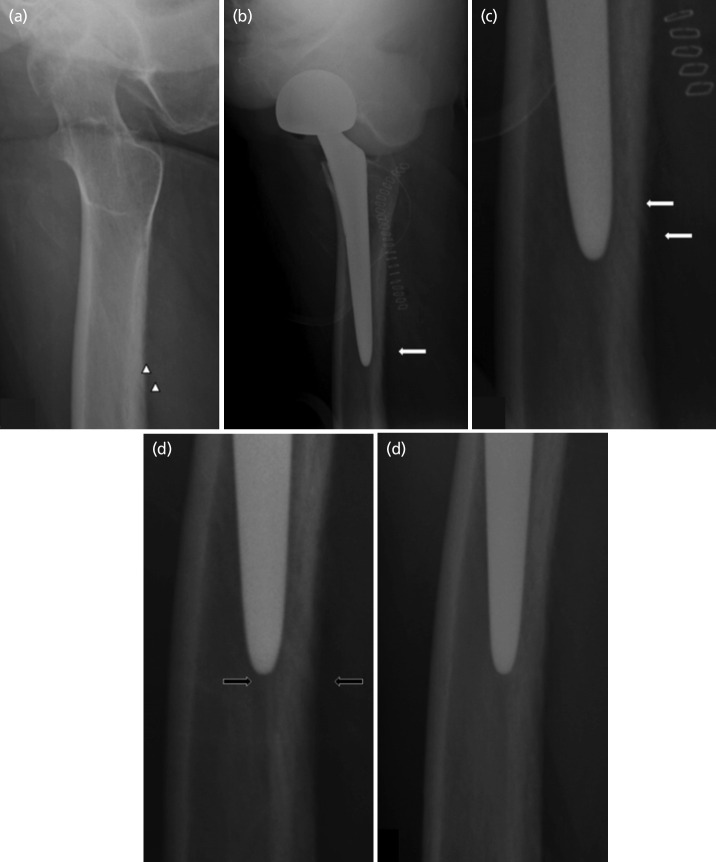
89 years old male patient with bipolar hemi arthroplasty. (a) Nutrient artery canal (arrowhead) by pre-operative translateral view. (b) Abnormal radiolucent line (arrow) by immediate post-operative translateral view. (c) Discontinuity and beak of posterior outer cortex (arrow) by immediate post-operative translateral view magnification. (d) At three months post-operative translateral view, the previous radiolucent line (black arrow) was faded and surrounding bony hypertrophy was visible. (3) At 15 months post-operative translateral view, the previous radiolucent was no longer visible.

Eventually, the three cases (1.2%, 3 of 242) had a fracture around the stem that were detected radiographically after THA. There was no specific abnormality during operation to suspect fractures in any of those cases. Most patients with THA start weight bearing exercises and staged rehabilitation program from the post-operative two weeks. According to the Lee *et al*^18^ patients with minimally displaced PFF of Vancouver type B successfully achieved bone union without surgical treatment but only with staged rehabilitation after four weeks of non-weight bearing. In our study, all patients were treated by conservative methods including non-weight bearing for three weeks post-operatively, partial weight bearing using crutches at four weeks and full weight bearing without assistance at six weeks. There was one notable case of a patient diagnosed with PFF that radiolucent line suspected of a fracture around the stem on immediate post-operative radiographs was in the same position as the NAC found in the pre-operative radiograph, but the discontinuity and beak of the posterior outer cortex were observed (Fig. 6a, b). On plain radiographs at post-operative three months, cortical hypertrophy was observed in the area suspected of the fracture. At post-operative 15 months, the radiolucent lines have completely disappeared confirming a complete union of the fracture (Fig. 6c, d, e). This case was noteworthy because the fracture occurred along the NAC was unlike other cases. These fractures are more difficult to diagnosis because the fracture lines overlap with the NAC. According to other literature, patients with severe osteoporosis, the NAC may be vulnerable to fracture^19^.

Under suspicion of PFFs, post-operative CT and bone scan can be performed for accurate diagnosis. Once PFF is confirmed, the treatment depends on the stability of the stem. Non-weight bearing ambulation can be attempted as a conservative treatment. Because Vancouver type B1 and B2 fractures are often reported to have poor prognosis in conservative treatment according to several literatures, it is recommended that the intra-operative PFF should be evaluated before closing the surgery^7,20^. The 'C-arm' mobile fluoroscopy can often miss undisplaced PFF due to poor resolution quality; therefore, patients with high risk of PFF should be checked using a portable radiograph image intensifier before closing the surgery.

On account of these features, we found several clues to differentiate the fractures from the NACs. The NACs are characterised by blunt ends, relatively smooth borders and sclerotic walls. They are also limited to one cortex and do not change in shape and course over time throughout follow-up radiographs. Fractures are characterised by relatively sharp borders and may involve both cortexes. If discontinuity of the outer cortex is visible, the fracture should strongly be suspected. On follow-up plain radiographs, formation of a callus or cortical thickening leads to a presence of a previous fracture. In addition, PFFs should be more strongly suspected if the radiolucent line is located in proximal or distal end of the implanted stem, starts from anterior cortex, or if the direction is transverse or downward.

There are several limitations to this study. One of the limitations includes its retrospective design and small size of the cases with fractures. Another limitation is that only the plain radiographs were assessed in the study. However, the utility of other imaging modalities such as bone scan, CT and magnetic resonance images in diagnosing fractures was beyond the scope of the current study.

## Conclusion

It is often difficult to distinguish between undisplaced PFF and NAC on post-operative plain radiographs in hip arthroplasty. We found that there were several different radiographic characteristics between NACs and PFFs. It is important to ensure in advance the position and shape of the NAC observed in the pre-operative radiographic images. If PFF is suspected, the radiolucent lines observed in preoperative and post-operative radiographic images should be carefully reviewed and compared, and additional examination such as CT or bone scan should be performed.
